# Supplementary material for: Impact of the Soweto football derby on the trauma emergency department at Chris Hani Baragwanath Academic Hospital, a tertiary level hospital in South Africa
Source: PLoS One. 2024 Jul 11;19(7):e0306836. doi: 10.1371/journal.pone.0306836 (PMC11239021; doi:10.1371/journal.pone.0306836)
Supplement: S1 Table — (DOCX) [file pone.0306836.s001.docx]

**S1 Table 1. Types of injuries seen during the Soweto Derby and the control**

| Type of injury | Blunt | Burns | Penetrating | Total |
| --- | --- | --- | --- | --- |
| Bite | 0 | 0 | 30 | 30 |
| Blunt assault | 648 | 0 | 0 | 648 |
| Blunt miscellaneous | 7 | 0 | 0 | 7 |
| Burns | 0 | 105 | 0 | 105 |
| Fall from height | 44 | 0 | 0 | 44 |
| Gun shot wound | 0 | 0 | 74 | 74 |
| Head injury | 26 | 0 | 11 | 37 |
| Motor bike accident | 4 | 0 | 0 | 4 |
| Motor vehicle accident | 350 | 0 | 0 | 350 |
| Pedestrian vehicle accident | 217 | 0 | 0 | 217 |
| Musculoskeletal | 486 | 0 | 30 | 516 |
| Stab | 1 | 0 | 433 | 434 |
| Train accident | 4 | 0 | 1 | 5 |
| Laceration | 0 | 0 | 81 | 81 |
| Total | 1787 | 105 | 660 | 2552 |
